# Multi-antigen chimeric antigen receptor-T cell therapy for relapsed/refractory B cell lymphomas

**DOI:** 10.3389/fimmu.2026.1900443

**Published:** 2026-07-22

**Authors:** Toshali Pandey, Divya Samat, Saurabh Dahiya, Shyam A. Patel

**Affiliations:** 1 Dept. of Medicine, Univ. of Arkansas College of Medicine, Little Rock, AR, United States; 2 Dept. of Medicine, Reading Hospital, Tower Health, Reading, PA, United States; 3 Division of Blood and Marrow Transplantation and Cellular Therapy, Stanford University School of Medicine, Stanford, CA, United States; 4 Dept. of Medicine – Division of Hematology/Oncology, UMass Memorial Medical Center, UMass Chan Medical School, Worcester, MA, United States; 5 Center for Clinical and Translational Science, UMass Chan Medical School, Worcester, MA, United States

**Keywords:** B cell lymphoma, CAR--t, immunotherapy, lymphoma, multi-antigen targeting, tandem CARs

## Abstract

In the wake of the 10-year anniversary since the introduction of chimeric antigen receptor-T (CAR-T) cell therapies to the market, the field of B cell lymphoma has seen remarkable advances since these therapies first arrived at the bedside in 2017. Modern data from longitudinal readouts attests to the high depth and durability of response to CAR-T therapies in many patients with B cell lymphomas; however, approximately 50% of patients continue to have relapsed/refractory disease even after receipt of conventional CAR-T constructs. In this review, we discuss the mechanisms underlying primary and secondary refractoriness to conventional single-targeting CAR-T therapies for B cell lymphomas and explore the ongoing challenges with existing CAR constructs. We discuss the prospects for adaptable multi-antigen targeting via the use of bivalent CAR and bicistronic CAR functionalities as informed by recent advances in synthetic immunobiology. We explore contemporary efforts, mostly Phase 1 and 2 trials, involving bivalent CAR and bicistronic CAR constructs at the cutting edge of clinical translation. Finally, we propose evidence-based solutions to help improve the translational success of multi-antigen CAR-T therapy, including optimizing construct architecture, expanding the targetable antigen landscape, and improving scalability. These solutions for multi-antigen targeting in next-generation CARs may have substantial benefits in the coming years.

## 1 Introduction

Chimeric antigen receptor-T cell (CAR-T) therapy has revolutionized the treatment of B cell non-Hodgkin lymphoma (NHL) in the past decade since its introduction to the commercial market in 2017. The success of CAR-T therapies has been preceded by well-informed pre-clinical work grounded in the disciplines of immunology and synthetic biology. As we approach the 10-year anniversary of CAR-T cell therapies, it is evident that remarkable advances have been seen across a variety of cellular therapy products. CAR-T therapies have been proposed to lead to deep and durable remissions with curative potential in aggressive B cell lymphomas (1, 2).

Seven CAR-T products are currently on the market and under active use, and four of these products are approved for the treatment of lymphomas. Axicabtagene ciloleucel (axi-cel) is a CD19-directed CAR-T cell therapy that was the first CAR-T product to receive Food and Drug Administration (FDA) approval for B cell non-Hodgkin lymphoma (1). The ZUMA-1 trial demonstrated an overall response rate (ORR) of 82% with a complete response (CR) rate of 54% in refractory large B cell lymphoma (LBCL) (1). Similar success was demonstrated by tisagenlecleucel (tisa-cel) in the Phase 2 JULIET trial for relapsed/refractory (R/R) LBCL and in follicular lymphoma in the Phase 2 ELARA trial (3, 4). Lisocabtagene maraleucel (liso-cel) achieved high success in R/R LBCL in the Phase 3 TRANSFORM trial and in chronic lymphocytic leukemia (CLL) in the TRANSCEND CLL 004 trial (5, 6). Brexucabtagene autoleucel (brexu-cel) achieved excellent results in mantle cell lymphoma in the ZUMA-2 trial and in B cell acute lymphoblastic leukemia (B-ALL) in the ZUMA-3 trial (7–9). Long-term follow-up data showed a median duration of response (DOR) ranging from 1 year to over 5 years depending on subtype of lymphoma and depth of response, attesting to long-term disease control for select recipients of CAR-T therapy (10).

While a subset of patients, particularly amongst those who achieved CR had very long (essentially curative) DORs, many patients experience resistance to CAR-T therapy in clinical trials and in real-world settings. This review discusses the fundamentals of the mechanisms of resistance and highlights tangible solutions via the use of multi-antigen CAR-T therapies.

## 2 Mechanisms of resistance to conventional CAR--T therapies

### 2.1 Overview of resistance mechanisms

Multiple mechanisms of resistance to CAR-T therapies have been described. Primary resistance occurs in a setting where there is lack of initial response or refractoriness to CAR-T therapy. Secondary resistance occurs in a setting of relapse after a first initial response (11). Resistance is common and happens in about half of patients with aggressive B cell NHL. A large observational study on outcomes of R/R LBCL after axi-cel treatment reported relapse in 49% of patients (of whom 28% had primary resistance) after a median follow up of 13 months (12). Similarly, a French observational study reported a relapse rate of 49% in aggressive B cell NHL treated with tisa-cel at median follow up of 8 months (13). Another study in the US showed a relapse rate of 55% at 18 months median follow up for R/R aggressive B-cell NHL treated with axi-cel, tisa-cel, or liso-cel (14). These high rates of resistance may reflect a selection pressure by the CAR-T product or may reflect inherent resistance at baseline.

Secondary resistance is defined as refractoriness that develops after exposure and response to the initial CAR-T therapy. Secondary resistance can be attributed to antigen-negative relapse, where the relapsed tumor lacks the original target antigen. Antigen-negative relapse is a common cause of secondary resistance in B-ALL; however, data from seminal clinical trials in B cell NHL are scarce (15–17). This is likely due to lack of baseline testing for target antigen expression in B cell NHL as well as reliance on immunohistochemistry (IHC) rather than flow cytometry for target antigen quantification. A two-year follow-up of the ZUMA-1 trial showed CD19 loss/reduction in 28% of relapsed cases where paired biopsies were available (18). Observational studies have shown rates of CD19 loss or downregulation ranging from 17% to 62% in post-CAR-T relapse biopsy samples that were assessed for CD19 in relapsed/refractory (R/R) aggressive B cell NHL suggesting a causative role in resistance (12, 14, 19, 20). These mechanisms are discussed herein.

### 2.2 Antigen escape

**Figure 1:**
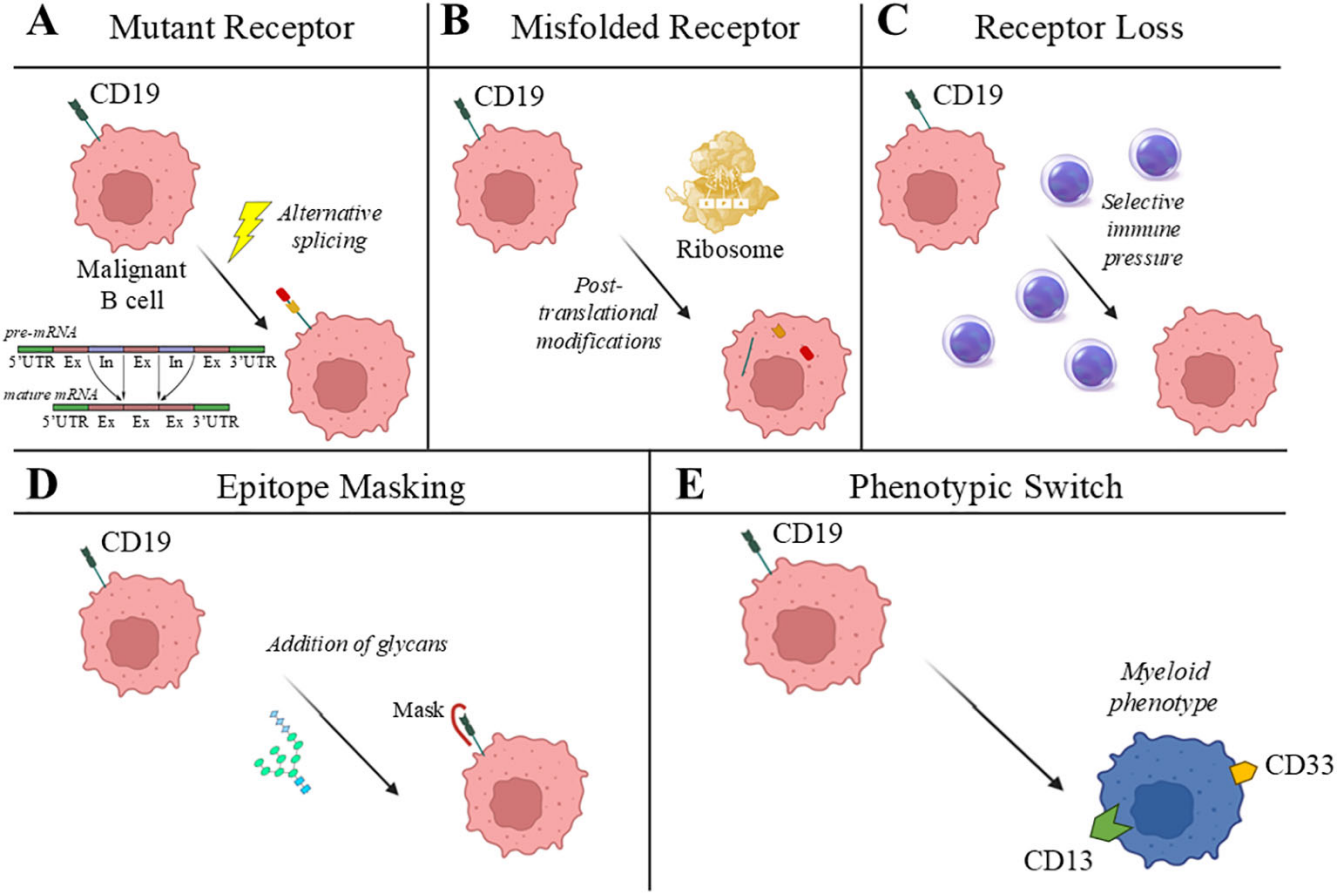
Mechanisms of resistance to conventional CAR-T therapies. Malignant B cells can evade CAR-T cells via **(A)** generation of mutant target receptors, **(B)** creation of misfolded receptors that escape detection, **(C)** gross loss of the receptor from the cell surface, **(D)** masking of the epitope known to be recognized by effector T cells, and **(E)** phenotypic switching toward a myeloid lineage. Figure was created with BioRender.

One of the major mechanisms of secondary resistance to CAR-T therapies is target antigen escape. Antigen-negative relapse is thought to be the result of antigen escape in the setting of selective pressure from CAR-T therapy. Several mechanisms of antigen escape have been described using data largely from B-ALL (11, 15–17, 21). Mutant receptors or antigens lacking the epitope recognized by CARs may arise when clones with alternative splicing pathways are selected (**Figure 1A**). For example, exon 2 or exon 4 skipping in CD19 has been described in patients with B-ALL, and these splice variants can alter the FMC63 epitope on CD19 (22, 23). Misfolded receptors/antigen are the result of alternative post-translational modifications that also result in unrecognizable epitopes (**Figure 1B**). Receptor loss occurs when immune pressure favors expansion of a B cell clone (pre-existing or acquired) that lacks the gene for the target antigen, presumably due to deletion (**Figure 1C**). Epitope masking is an unusual but known form of antigen-negative relapse due to the inadvertent transduction of malignant B cells resulting in a product that binds to and prevents recognition of the target antigen (**Figure 1D**) (21).

Detection of antigen escape might be performed via IHC, flow cytometry, or next-generation sequencing (NGS), and each has its advantages and limitations. For example, flow cytometry is readily accessible but faces technical hurdles in processing lymphoma tissue samples in clinical practice. IHC may be associated with quicker turnaround time and lower costs, with lower sensitivity. NGS may be associated with longer turnaround time and higher costs, with higher sensitivity. Standardization of antigen escape detection is needed.

### 2.3 Lineage switching

Another mechanism of resistance, though less common, is lineage switching. This phenomenon can be seen in mixed phenotypic leukemias where the leukemia cells shift from a B cell lineage to a myeloid lineage. This results in loss of expression of the target antigen. Lineage plasticity underlies resistance to CAR-T therapies that target a specific antigen (24). B cell markers such as CD19 and CD20 may be downregulated, while myeloid markers such as CD13 and CD33 can be upregulated (**Figure 1E**) (25). Outcomes of patients with high lineage plasticity tend to be dismal. Lineage plasticity also includes adaptive antigen modulation, which can impair the efficacy of conventional CAR-T therapies directed against CD19 in B cell lymphomas.

## 3 Strategies for multi-antigen targeting

### 3.1 Overview of strategies

The conceptualization and implementation of multi-antigen CAR-T therapy was driven by the development of antigen escape and lineage switching arising as a result of selective immune pressure for a single antigen. By targeting more than one antigen at a time, multi-antigen CAR-T cells offer the chance to avoid resurgence of disease from a single antigen-negative clone. One of the first strategies in multi-antigen targeting that showed success was in the setting of pediatric B-ALL (26). In a multi-center Phase 2 trial, co-administration of CD19 and CD22-directed CAR-T cell therapy showed a CR rate of 99% in children (26). Several strategies for multi-antigen targeting are currently under investigation, with variable success seen in early phase trials. Herein, we focus on practical and adaptable multi-antigen CAR-T approaches for adult B cell NHL, including the leading products in development.

**Figure 2:**
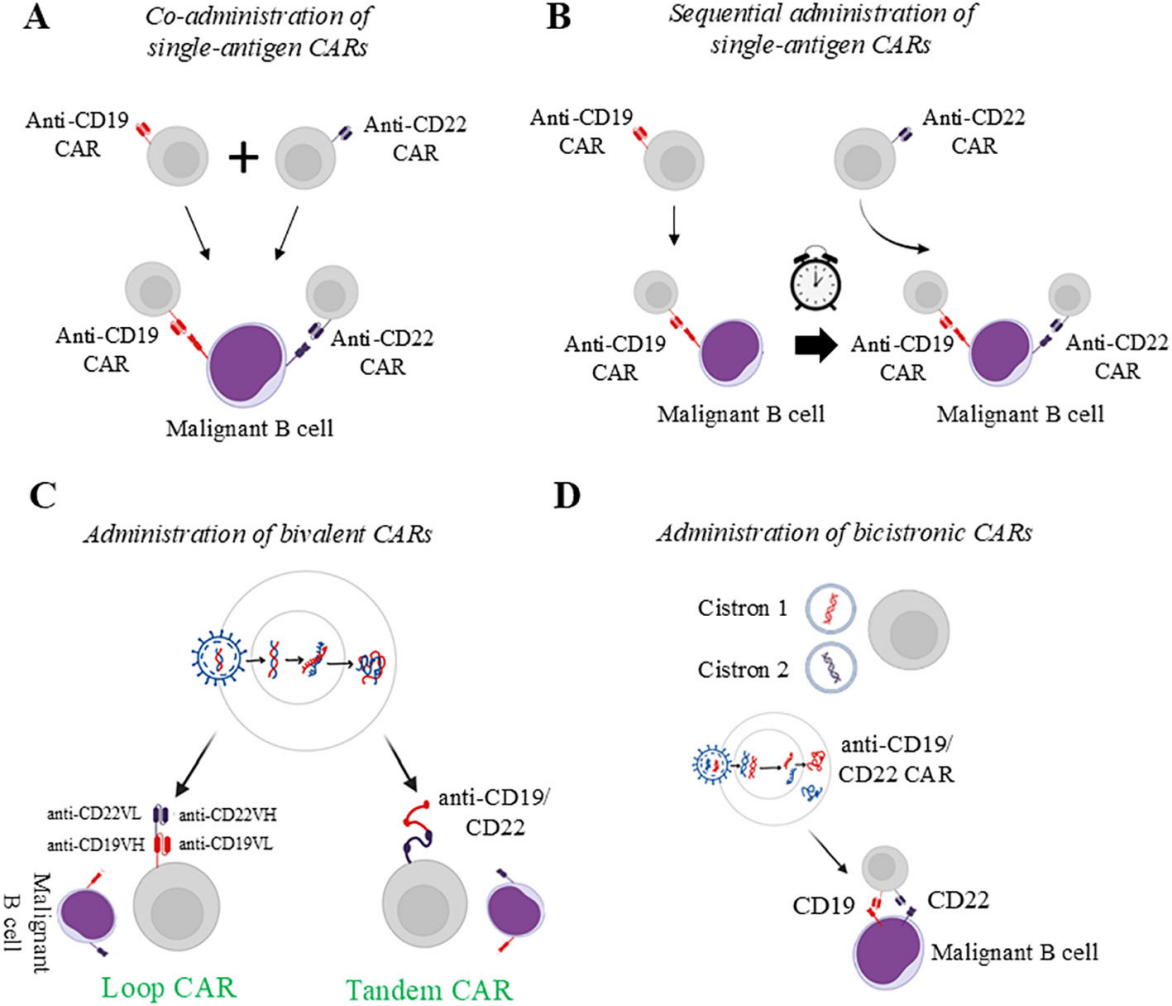
Multi-antigen CAR-T delivery modalities and construct design. Delivery modalities include **(A)** concurrent administration of two separate single antigen-targeting CAR-T constructs in different cells (aimed at the malignant B cell), **(B)** concurrent administration of two separate single antigen-targeting CAR-T constructs in different cells, **(C)** administration of bivalent CAR-T cells (including loop design and tandem design), and **(D)** administration of bicistronic CARs harboring two distinct genetic components leading to two separate CARs. Figure was created with BioRender.

There are a number of different methods for administration of multi-antigen CAR-T cells. Co-administration involved two or more CAR-T products, encoding receptors for different target antigens, are first manufactured separately, then infused as a pooled product (**Figure 2A**). In some cases, there may be a time interval between infusion of these two separate products (sequential administration) (**Figure 2B**). A disadvantage of such a strategy would be the elevated cost and risk of failure due to multiple rounds of manufacturing and generation of two or more vectors. Furthermore, co-administration can lead to a heterogeneous population of CAR-T cells with some expressing both CARs and some expressing either of the two CARs.

### 3.2 Configurations of multi-antigen CARs

Broadly, the configurations of multi-antigen CARs include bivalent CARs and bicistronic CARs (27). Within the category of bivalent CARs, the two most common subtypes include loop CARs and tandem CARs (**Figure 2C**). Loop CARs harbor a unique conformational design that fuses together the binding domains of two separate antibodies, and this technology allows for simultaneous binding of two separate antigens. Tandem CAR-T cells, on the other hand, are bivalent CARs that contain two single-chain variable fragments connected in continuity (in “tandem”), and these CARs target two different antigens. In a tandem CAR-T design, the vector contains one genetic sequence that contiguously encodes for a CAR containing two or more heavy/light chain fragments with different antigen specificity. Tandem CARs incorporate two different antigen-recognition domains, which allow for capture of diverse target antigens. The structure of the tandem CAR molecule has a major impact on its ability to target two antigens simultaneously and requires precise engineering that leads to a single peptide. The main value of loop and tandem CARs is that they bypass the issue of selective pressure that contributes to single antigen-negative relapse.

Bicistronic CARs constitute a unique modality in which a vector contains two separate and complete sequences (cistrons) and thereby two different CARs on the same cell. These two sequences are separated by a self-cleaving peptide. This results in a homogeneous population of CAR-T cells that express both CARs on each T cell, and each of these CARs can uniquely bind to a separate antigen (**Figure 2D**). The classic antigen pair for bicistronic targeting is CD19/CD22 in B cell lymphomas (28). Due to the longer length of the bicistronic sequence compared to a mono-specific CAR, a limiting factor for manufacturing is the ability of the vector to successfully carry the sequence. Bicistronic CAR designs have also been attempted in solid tumors, such as the use of anti-EGFR/anti-IL13Rα2 in glioblastoma (29).

There are functional differences between bivalent and bicistronic CARs in terms of T cell activation intensity, cytokine secretion profiles, and off-target risks. Preclinical studies have suggested that bicistronic CARs are superior to bivalent CARs because they often lead to improved clearance of the target antigen and reduced T cell exhaustion (30). This is likely due to dual co-stimulation. However, bicistronic CARs have lower transduction efficiency and larger vector size, while bivalent CARs have lower manufacturing costs.

### 3.3 Targets for multi-antigen CAR constructs

Pan-specific B cell markers are attractive targets for B cell lymphomas given their lineage-specific expression and accessible surface location. CD19 holds the strongest track record as a CAR-T target for B cell lymphomas, but other lineage-specific antigens have been studied and may be promising. CD20 is an established marker of the B cell lineage, and its therapeutic utility as a target is best exemplified by rituximab, which forms the backbone of most B cell NHL treatment regimens. CD22 is a regulator of B cell receptor function and has been demonstrated as a highly sensitive and specific marker for B cells (31, 32). There are two antigen-drug conjugates (ADCs), inotuzumab ozogamicin and moxetumomab pasudotox that are CD22-directed and have received FDA approval for B-ALL and hairy cell leukemia, respectively (32–34). Another promising pan B-cell marker is CD79b against which the ADC polatuzumab vedotin has shown significant success in diffuse large B cell lymphoma (DLBCL) (35). All these antigens are being actively studied as CAR-T targets in B cell NHL, and many of these studies involve combinatorial strategies, as noted below (36).

Antigen-pair selection rationale is highly relevant in the era of multi-antigen CAR-T therapies. For example, there may be notable differences in response rates for anti-CD19/CD20 vs. anti-CD19/CD22 targeting. CD22-negative relapse occurred in 53.8% of AUTO3 recipients, while dual-negative CD19/CD20 loss was documented in <5% of CD19/CD20 trial relapses (28). This suggests CD20 may confer greater protection against antigen-negative relapse than CD22. An understanding of mechanisms of antigen escape may guide the selection of antigen pairs. A recent study on bispecific CAR-T cells directly addressed this axis and provided relevant comparative context on antigen escape profiles after multi-antigen targeting (37). This study showed the value of structure-based optimization alongside intracellular modulation to improve CAR-T persistence and improve durability.

## 4 Key clinical trials for multi-antigen car-T cell therapies

### 4.1 Overview

Various clinical trials are currently underway for multi-antigen targeting (**Table 1**). Of note, follow-up durations of these active trials vary substantially (ranging from 3 months to 30 months), and direct cross-comparison of ORR and CR rate may lead to biased conclusions, so this is a limitation of interpretation. One of the first multi-targeting CAR-T therapies for B cell NHL evaluated was the bivalent CD19/CD22-directed CAR in a Phase 1 trial, led by Stanford, involving patients with R/R LBCL and B-ALL (38). Of the 21 R/R LBCL patients, the best ORR was 62% with a CR of 29% and a modest median progression-free survival (mPFS) of 3.2 months. During the development of this autologous CD19/22 dual-targeting CAR-T, Qin and colleagues evaluated a series of multi-targeting approaches, including co-administration, sequential administration, co-transduction and tandem CARs (27). The final product, LoopCAR6, was a tandem CAR that had a short linker between the CD19 single-chain variable fragment (scFv) and CD22 variable chains that had the best target detection and IL-2 production rates against leukemic cells expressing only one out of the two target antigens. The major active trials are discussed below.

**Table 1:** Summary of active clinical trials for multi-antigen targeting CAR-T cell therapy in B cell lymphomas.

Author/year	Trial	Phase	CAR construct	Target antigens	Disease	Median follow up (months)	ORR	CR	Response (months, unless specified)	Toxicity	Relapse
Borchmann et al., 2025 (40)	DALY 2-EU (NCT04844866)	2	Zamtocabtagene autoleucel; dual-targeting autologous tandem CAR-T (MB-CART2019.1)	CD19/20	R/R LBCL	17	72%	54%	mEFS 6.2; mPFS 8.5	Gr ≥3 CRS: 5.3% (4 pts); Gr ≥3 ICANS 1.3% (1 pt)	
Dahiya et al., 2025 (44)	NCT04989803	1	KITE-363; dual-targeting bicistronic autologous CAR-T	CD19/20	R/R B cell lymphoma	11.1	For CAR-naïve subset only (n=23), 87%	For CAR-naïve subset only (n=23), 78%	OS rate 76% (across DL1-DL3)	Gr ≥3 CRS: 2.7% (1 pt); Gr ≥3 ICANS 8% (3 pt)	
Dahiya et al., 2025 (44)		1	KITE-753; dual-targeting bicistronic autologous CAR-T	CD19/20	R/R B cell lymphoma	4.4	71% (across DL1-DL3)	57% (across DL1-DL3)		Gr ≥3 CRS: 7% (1 pt); Gr ≥3 ICANS 0%	
Larson et al., 2025	NCT05826535	1/2	Rondecabtagene autoleucel, dual targeting tandem autologous CAR-T (LYL314, IMPT-314)	CD19/20	R/R LBCL	7-10*	87% (2L setting); 90% (3L+ setting)	100% (2L setting); 71% (3L setting)		Gr ≥3 CRS: 0%; Gr ≥3 ICANS 13% (8/60)	
Patel et al., 2025 (41)	NCT05421663	1b	Prizloncabtagene autoleucel, dual-targeting tandem autologous CAR-T (JNJ-90014496)	CD19/20	R/R LBCL	6	91%	75%		Grd ≥3 CRS: 4% (2); Grd ≥3 ICANS 8% (4)	
Roddie et al., 2023 (28)	ALEXANDER (NCT03289455)	1	AUTO3, dual-targeting bicistronic autologous CAR-T	CD19/22	R/R LBCL	21.6	66%	49%	mDOR 8.3, mPFS 3.32, mOS 13.8	Gr ≥3 CRS: 1.9% (1); Gr ≥3 ICANS 3.8% (2)	CD19(-) 15%, CD22(-) 53.8%
Shah et al., 2024 (9)	DALY II USA (NCT04792489)	2	Zamtocabtagene autoleucel; dual-targeting autologous tandem CAR-T	CD19/20	R/R DLBCL	3 (minimum)	73%	49%	12-mo PFS 42%, 12 mo OS 72%, mDOR 11.4	Gr ≥3 CRS: 0%; Gr ≥3 ICANS 4.3% (3)	Of 24 relapses, CD19(-) was in 2, CD20(-) was in 3, double neg in 1 patient
Spiegel et al., 2021 (38)		1	LoopCAR6; dual-targeting tandem autologous CAR-T	CD19/22	R/R LBCL	10	62%	29%	mPFS 3.2, mOS 22.5	Gr ≥3 CRS: 4.7% (1); Gr ≥3 ICANS 4.7% (1)	29% CD19(-) relapse, CD22 preserved
Vasu et al., 2024 (49)	NCT05418088	1	DuoCAR20.19.22D9; trispecific autologous tandem bispecific and a monospecific CAR-T	CD19/20/22	R/R B cell NHL		50%	44% (NHL)		Gr ≥3 CRS: 0%; Gr ≥3 ICANS 0%	1 had reduction in CD20 expression, 1 converted from CD20(-) to (+)
Yu et al., 2025 (45)	NCT05149391, NCT04317885, NCT04655677, NCT04696432, NCT04693676	1	Prizloncabtagene autoleucel, dual-targeting autologous tandem CAR-T (C-CAR039)	CD19/20	R/R B cell NHL	30	91%	86%	2-year DOR 68.1%, PFS 62.6%, OS 76.5%	Gd ≥3 CRS: 2% (1); Grd ≥3 ICANS 0%	Of 6 relapsed patients, 1 had CD19 loss, one had dual CD19/CD20 loss
Zhang et al., 2022 (48)	NCT03097770	1/2	TanCAR7; dual-targeting tandem autologous CAR-T	CD19/20	R/R B cell NHL	27.7	78%	70%	12-mo ORR 76%, mPFS 27.6, mOS not reached	Gr ≥3 CRS: 10% (9); Gr ≥3 ICANS 2.3% (2)	1 out of 16 relapsed patients had a CD19/20 dual loss. No CD19(-) neg or CD20(-) relapses
Qu et al., 2022 (50)	NCT03196830	2	Dual-targeting tandem autologous CAR-T	CD19/22	R/R DLBCL	10.9	91%	64%	mPFS 10.2, mOS NR	Gr ≥3 CRS: 21% (7); Gr ≥3 ICANS 0%	

DL, dose level; DLBCL, diffuse large B cell lymphoma; DOR, duration of response; EFS, event-free survival; Gr, grade; m, median; NHL, non-Hodgkin lymphoma; NR, not reached; OS, overall survival; PFS, progression-free survival.

*7 months for the 2^nd^ line setting; 10 months for 3^rd^ line setting; survival data remains unpublished

### 4.2 Zamtocabtagene autoleucel (zamto-cel)

Zamto-cel is an autologous tandem CD19/20-directed CAR-T and has been studied in two Phase 2 multi-center RCTs in Europe and the United States. Shah et al. presented pre-planned interim results from the single-arm DALY II USA trial evaluating zamto-cel as third-line therapy for R/R DLBCL (39). Of the 59 patients who were response-evaluable, the ORR was 73%, with a CR rate of 50% and a 12-month PFS of 42%. Shortly after this study, Borchmann et al. reported on the DALY 2-EU trial comparing zamto-cel with rituximab/gemcitabine/oxaliplatin (R-GemOx) as second-line therapy for R/R LBCL (40). This is one of the largest randomized controlled trials for multi-targeting CAR-T in B cell NHL. The median event-free survival (EFS) was 6.2 months for zamto-cel (n=82) compared with R-GemOx (n=86) with a hazard ratio (HR) of 0.39. Median PFS was significantly longer with zamto-cel as well (8.5 months vs 3.3 months) with an ORR of 72% in the intention-to-treat arm and a favorable safety profile.

### 4.3 Rondecabtagene autoleucel (ronde-cel)

Ronde-cel is another autologous tandem CD19/20-directed CAR-T that is manufactured from CD62L(+)-enriched naïve and central memory T cells (41). Enrichment for CD62L(+) was with the intention to reduce T cell exhaustion and to enhance persistence. Analysis of the ronde-cel product revealed increased expression of memory-related genes IL-7R and CCR7 (in addition to CD62L which was expressed by 95% cells in the product) and lower expression of exhaustion-associated genes (TIGIT, PD-1, and LAG3) compared to an approved CD19 CAR-T therapy (42). Larson et al. tested ronde-cel in the high-risk second-line beyond setting for R/R LBCL. Of the 15 patients in the second-line setting, the ORR was 87% and CR rate was 60%. These results were replicated in the third-line and beyond setting with ORR of 90% and CR rate of 71% (41). Survival data have not been published yet. Notably, the high efficacy achieved by ronde-cel was with zero grade 3 or higher cytokine release syndrome (CRS) events. This may reflect a greater selectivity and efficiency conferred by the more memory-like phenotype of the product. A Phase 3 head-to-head randomized controlled trial known as the PiNACLE-H2H comparing ronde-cel with CD19 CAR-T is currently underway.

### 4.4 KITE-363 and KITE-753

KITE-363 and KITE-753 are both autologous bicistronic CD19/20-targeting CAR-T cells withunique features. The *ex vivo* expansion period of KITE-753 was deliberately shortened to enable an increased proportion of naive T cells, hypothesized to have a more durable performance and a shorter turnaround time. Investigators were able to demonstrate anti-tumor activity with KITE-753 at a 25-fold lower dose (43). Dahiya et al. reported results of a Phase 1 trial using both products in 59 patients with R/R LBCL, 43% of whom had prior CAR-T exposure (44). At a dose of 2.0 x10^5 CAR-T cells/kg, KITE-753 demonstrated a CR rate of 100%, though this interpretation is limited by the fact that this was a single-dose level with a median follow-up of only 4.4 months. KITE-363 had an ORR of 87% among CAR-naïve patients (CR rate 78%). Given the nascent data, it will be challenging to make meaningful or definitive conclusions without longer-term readouts and a higher sample size.

### 4.5 Prizloncabtagene autoleucel (prizlon-cel)

Prizlon-cel is a CD19/20-targeting tandem autologous CAR-T product under investigation (45). The anti-CD20 scFv of prizlon-cel recognizes two epitopes that are distinct from those recognized by rituximab, theoretically allowing prizlon-cel to be effective in CD20(+) relapse. In a Phase 1 multi-center RCT in China, of the 47 evaluable patients with R/R B cell NHL who received prizlon-cel in the third-line and beyond setting, the ORR was 91.5% with a CR rate of 85.1% and a 2-year PFS of 62.6%. This led to an ongoing global trial, the results of which were presented by Patel et al. at Society of Hematologic Oncology conference in 2025 (46). Of the 44 response-evaluable patients with R/R LBCL, the ORR was 91% with a CR rate of 75%.

### 4.6 AUTO3

AUTO3 is one of the first CD19/22 targeting bicistronic autologous CAR-T cell products. It was initially studied in the pediatric AMELIA study involving patients with R/R B cell ALL with favorable initial response rates, but poor CAR-T persistence (47). To improve persistence, Roddie et al. studied AUTO3 in combination with checkpoint inhibition (pembrolizumab) in patients with R/R LBCL in the Phase 1 ALEXANDER trial (28). The duration of detectable CAR-T by quantitative polymerase chain reaction was a median of 50 days. At a median follow-up of 21.6 months, 66% of the 47 evaluable patients had an objective response, with a CR rate of 49%. They noted that neither AUTO3 dose nor pembrolizumab dose/timing were correlated with response, whereas low disease burden prior to lymphodepletion did.

### 4.7 TanCAR7

TanCAR7 is a tandem autologous CD19/20 targeting CAR-T product that was studied in patients with R/R B cell NHL (48). A total of 87 patients received TanCAR7 in this Phase 1/2 study, of which 78% had an objective response and 70% had a CR. The ORR was similar across tumor subtypes and prognostic subgroups. Sixty percent of patients remained in remission at a median follow-up of 28 months. The duration of response appeared to be affected by performance status, disease burden at enrollment, and extranodal disease. Six of 9 patients with prior CAR-T therapy achieved a CR.

### 4.8 Other multi-antigen CAR-T cells

Of the handful of studies utilizing more than two target antigens in B cell NHL, Vasu et al. presented results of a first-in-human Phase 1 study at the 2024 American Society of Hematology (ASH) conference (49). The product, duoCAR20.19.22D94, is an autologous tri-specific CAR-T that targets CD19, CD20, and CD22. This tri-specificity is conferred through a combination of a tandem CD19/20 CAR and a monospecific CD22 CAR on each T cell. The CD19/20 CAR also has a OX40 co-stimulatory domain postulated to enhance persistence and expansion. Given the complexity of the CAR-T product, it is notable that the median transduction efficiency stood at 26% and manufacturing was successful in 15 of 16 patients. Of the nine patients with R/R B cell NHL, five patients achieved CR, three of which persisted beyond one year.

Tandem autologous CD19/22 CAR-T has also been studied with the use of decitabine as part of the lymphodepleting regimen prior to CAR-T infusion (50). The investigators postulated that decitabine would lead to epigenetic alteration of tumor suppressor genes and enhance CD19 expression on lymphoma cells, thus leading to reduced tumor growth and greater target recognition. In their Phase 2 study, 33 patients with R/R DLBCL received decitabine in addition to fludarabine and cyclophosphamide. With a median follow-up of 11 months, results showed an ORR of 94% and a CR rate of 64%. The 2-year PFS and OS rates stood at 47.2% and 54.3%, respectively.

## 5 Future directions for development of multi-antigen CAR--T cells

### 5.1 Establishing superiority over single-antigen CAR-T therapies

The most pressing unresolved question is whether multi-antigen targeting confers a clinically meaningful advantage over existing CD19-directed CAR-T therapies. The current evidence base consists almost entirely of single-arm studies. The DALY 2-EU trial compared zamto-cel with R-GemOx rather than to an approved CD19 CAR-T product (40). The PiNACLE-H2H randomized trial comparing ronde-cel directly against a CD19-directed CAR-T will be the first to address this question, hopefully definitively, and future pivotal trials should be designed with this comparison as a primary objective. Until head-to-head data demonstrate a clear advantage in EFS or antigen-negative relapse prevention, the incremental benefit of multi-antigen targeting over the existing standard remains debatable. As such, Phase 3 trials involving multi-antigen CAR-T therapy have practice-changing potential.

### 5.2 Selection and optimization of bivalent vs. bicistronic architecture

The choice between bivalent and bicistronic architecture has translational consequences, including implications for CAR-T persistence *in vivo*. As an example, the poor persistence of CAR-T cells in AUTO3 from the AMELIA study might be explained by vector silencing or unbalanced surface expression (28, 47). The poor persistence from AUTO3 may also be related to the constant signaling from two independent CAR receptors, which may cause T cell exhaustion. T cell exhaustion can, in turn result in differentiation into short-lived effector cells. On the other hand, bivalent CARs, such as zamto-cel, ronde-cel, prizlon-cel, and TanCAR7, have their own disadvantages, such as steric hindrance (spatial blocking), eventually resulting in aberrant signaling leading to premature T cell exhaustion. This might account for their limited efficacy. Manufacturing constraints may need to be optimized in the upcoming years to help mitigate the risk of T cell exhaustion.

### 5.3 Standardizing antigen escape assessment

A fundamental gap is the absence of standardized antigen reassessment at relapse. Baseline biopsy rates across the trials reviewed above are low, and IHC remains the predominant tool despite being less quantitative than flow cytometry. There is no consensus on timing of re-biopsy. The field may benefit from appraisal of the antigen landscape at baseline for the purposes of response prediction. Correlative science in the field of multi-antigen targeting may involve the optimal use of biomarkers to predict response to cellular therapies. The most practical biomarkers for response are cell surface antigens on malignant B cells, as these are readily accessible and generally easy to detect via conventional means. Baseline biopsies prior to administration of multi-antigen CAR-T therapies might guide therapeutic decisions, based on the antigen repertoire present within each patient’s unique tumor. Building systematic, protocol-mandated antigen assessment into future trial designs will be highly helpful for the field to accurately quantify the efficacy of multi-antigen targeting in preventing antigen-negative relapse.

### 5.4 Expanding the repertoire of targetable antigens

**Figure 3:**
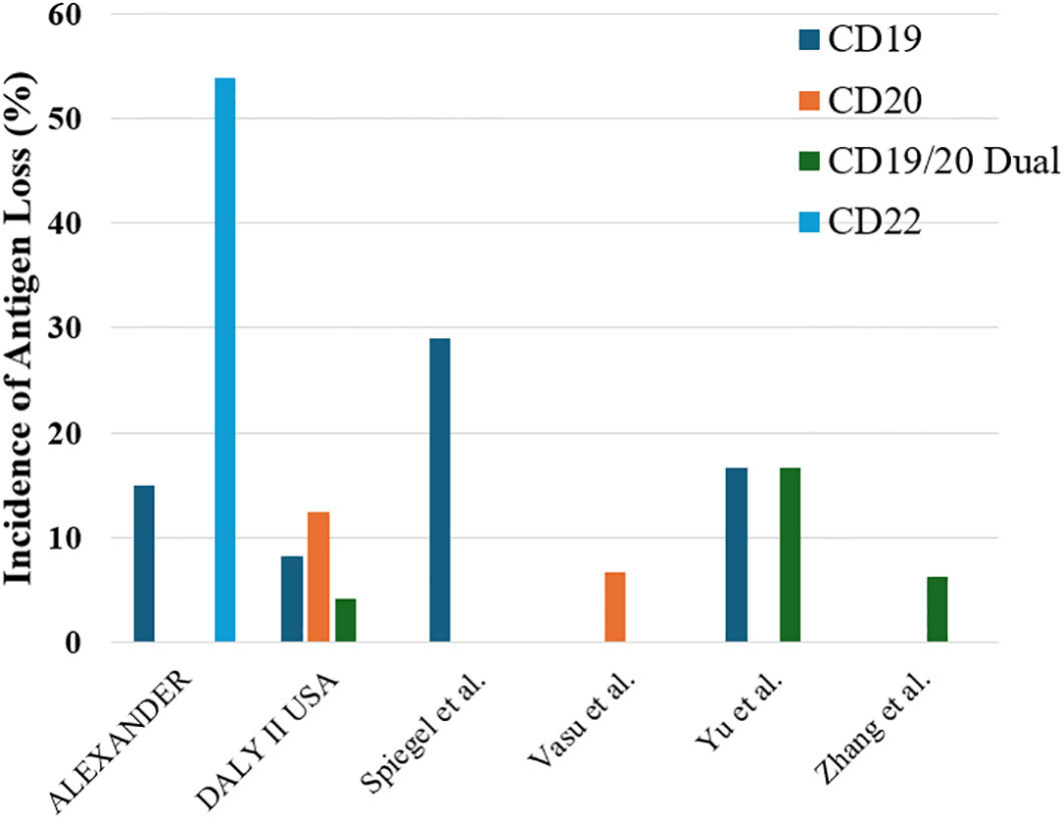
Incidence of antigen loss across key clinical trials for multi-antigen CAR-T therapies.

Antigen loss is a potential barrier to the field of multi-antigen targeting (**Figure 3**), and the field remains heavily concentrated on CD19, CD20, and CD22 as target antigens. Whether CD19/20, CD19/22, or tri-specific combinations are superior remains an open clinical question, given that CD20 operates independently of CD19 regulatory pathways while CD22 provides high sensitivity as a B cell lineage-defining marker. Beyond these, CD79b is a natural next candidate: CAR-T cells directed against CD79b have demonstrated robust preclinical activity, including against CD19-negative lymphoma (51, 52). CD37 is another promising target: CAR-37 T cells produced responses in four of five patients in the first-in-human trial, including three CRs, but were associated with severe and prolonged pancytopenia that will require careful engineering solutions before clinical incorporation (53). Other emerging pan-B cell targets including ROR1, BCMA, and BAFF-R deserve systematic exploration (54, 55). BCMA has been validated as a target antigen across a variety of B cell lymphomas including LBCL, MCL, FL, and CLL (55). Tri-specific CARs represent an important proof of concept, but whether a third antigen confers incremental benefit over dual-targeting requires validation in larger studies (49).

### 5.5 Optimizing construct architecture

Although novel CAR-T construct designs have gained traction, no clinical trial has directly compared bivalent (tandem or loop) vs. bicistronic architectures. As described above, preclinical evidence points to meaningful differences in co-expression consistency, manufacturing yield, and anti-tumor potency, but how these distinctions translate to clinical outcomes in NHL remains unknown (27). In an ideal situation, Phase 3 randomized control trials would evaluate response metrics for conventional CAR-T cells vs. multi-antigen CAR-T cells, with subgroup analyses for different modalities of multi-antigen targeting. Phase 1 trials have been promising, but the field does not know the optimal construct architecture that leads to the most superior responses.

**Figure 4:**
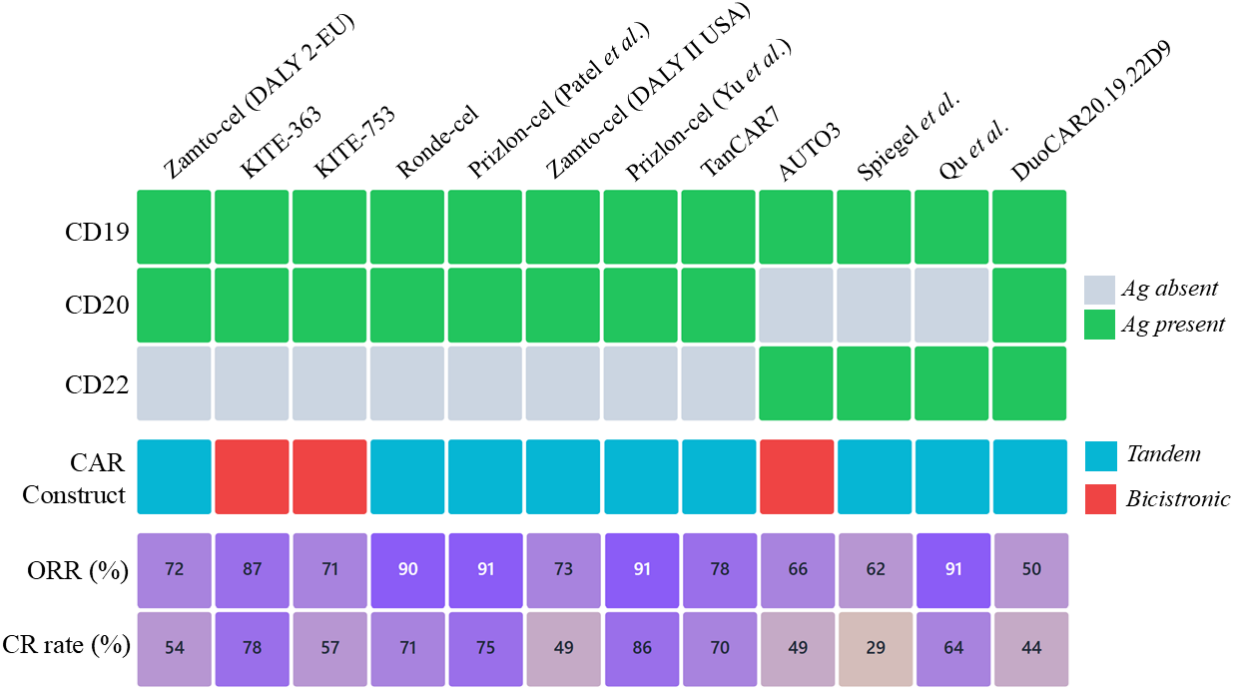
Summary of antigen-pair design and CAR vectors across key clinical trials for multi-antigen CAR-T cells. Response rates are shown. Abbreviations: ORR, overall response rate; CR, complete response.

Logic-gated technology can be incorporated into bivalent or bicistronic CARs to allow for improved adaptability and precision attacks on target cells (56). Logic gating typically involves the integration of multiple signals by the CAR-T cells prior to a decision being rendered about whether or not to attack the target cell. These CAR-T cells use Boolean logic, including functions such as “AND,” “OR,” or “NOT.” The “AND” logic gate permits the multi-antigen CAR-T cell to detect and eliminate the malignant cell when both target antigens are present. The SynNotch system uses AND gating and involves the binding of a primary antigen to a synthetic Notch receptor (57). The first antigen drives the expression of a classical CAR directed against the second target antigen. Thereafter, the CAR-T cells can only recognize and kill targets in the presence of both antigens. The “OR” logic gate, in contrast, permits for malignant cell destruction when either of the antigens is present, such as either CD19 or CD22. The “NOT” gate permits the CAR-T cell to inactivate in the setting of the presence of a healthy marker; an inhibitory signal results in lack of CAR-T activation as a safety measure to avoid autoimmunity. Optimization of construct architecture might be informed by long-term readouts of the contemporary investigational constructs in ongoing trials (**Figure 4**).

### 5.6 Overcoming T cell dysfunction

CAR-T persistence and exhaustion remain central determinants of durable response. One of the major mechanisms of resistance to conventional CAR-T therapies is T cell exhaustion (58, 59). Converging strategies address these T cell-specific challenges from different angles. Examples include the use of CD62L(+) T cell enrichment to preserve a stem-like phenotype (ronde-cel), shortened *ex vivo* expansion to reduce differentiation (KITE-753), and CRISPR-mediated knockout of inhibitory receptors such as PD-1 and TGFβRII to bypass microenvironmental suppression (42, 60, 61). A more compelling strategy appears to be CAR-T cells “armored” against T cell dysfunction and suppression by the tumor microenvironment. Many strategies have been developed starting with the constitutive secretion of stimulatory cytokines (such as IL-12, IL-18, IL-7), constitutive expression of CD40L by CAR-T cells and newer strategies such as expression of “decoy” TGF- β receptors (62).

Recently, Svoboda et al. published results of a seminal Phase 1 trial examining huCART19-IL18 in a heavily pre-treated R/R B-cell NHL population (20 out of 21 patients had failed prior anti-CD19 CAR-T therapy) (63). This product was a fourth-generation armored CAR-T with constitutive secretion of IL-18, and an expedited 3-day manufacturing process to enrich for less-differentiated CAR-T cells. Remarkably, huCART19-IL18 achieved an 81% ORR specifically in patients who had failed prior CD19 CAR-T therapy, with durable responses beyond two years (63). The integration of such armoring strategies into multi-antigen constructs represents a logical next step, though the added engineering complexity and potential for cytokine-mediated toxicity require careful evaluation (64).

### 5.7 Improving manufacturing scalability and access

Bivalent and bicistronic constructs present inherently greater manufacturing challenges than monospecific counterparts, including longer vector sequences, variable transduction efficiency (as low as 26% in the trispecific duoCAR product) and complex quality control requirements (49). Point-of-care manufacturing and shortened *ex vivo* expansion are promising approaches to reduce turnaround time and cost, but their scalability across decentralized networks has not been established (65, 66). Trial populations to date have been highly selected, and whether the efficacy advantages of multi-antigen targeting justify added manufacturing complexity in a real-world setting remains to be seen. Addressing this question will ultimately determine clinical uptake of multi-antigen CAR-T therapies across global markets.

Allogeneic off-the-shelf multi-antigen CAR-T cells represent a core solution to reduce manufacturing costs and decrease production turnaround time, and durable remissions have been observed in case series (67). Allogeneic multi-antigen CAR-T-based products have shown success in the B-ALL space (combination of allogeneic CD19/22 targeting CAR-T and an engineered allo-transplant graft called Orca-T to enhance allogeneic CAR-T persistence) (68). The ANTLER study is a Phase 1 trial in R/R B-cell NHL evaluating vispa-cel, an allogeneic CRISPR-edited (TCR and PD-1 knockout) anti-CD19 CAR-T product that had an impressive 94% ORR and a 69% CR rate (61). While studies on allogeneic multi-antigen CAR-T cells in the B cell NHL space are yet to publish data, a promising candidate appears to be P-CD19CD20-ALLO1. This is a CD19/20 targeting, allogeneic T stem cell-rich product that has shown pre-clinical success and is now being evaluated in Phase 1 trial on R/R B cell malignancies (NCT06014762) (69). Inducible pluripotent stem cell-derived platforms and point-of-care manufacturing (involving allogeneic products) may also improve scalability.

### 5.8 Extending the therapeutic reach across various NHL subtypes

The bulk of available data for multi-antigen targeting pertains to LBCL. Antigen expression and the tumor microenvironment differ meaningfully across follicular lymphoma, mantle cell lymphoma, and marginal zone lymphoma (4, 7). Disease-centric trials are needed to determine whether multi-antigen CAR-T strategies are equally effective across the spectrum of B cell NHL. The inclusion of more rare subtypes of lymphoma, such as primary mediastinal LBCL or gray-zone lymphoma, will be valuable to help expand therapeutic prospects of multi-antigen targeting to these subtypes of lymphomas. Given the efficacy of conventional CAR-T therapies in follicular lymphoma and chronic lymphocytic leukemia, expanding the therapeutic reach across indolent lymphomas may be a promising next step.

## 6 Conclusions and limitations

The landmark trials for conventional CAR-T cells have led to unprecedented success in the treatment of B cell lymphomas over the past decade, but the development of resistance to these CAR-T therapies (based on the mechanisms described above) has become a substantial pressing issue. This spurred interest in novel modalities of CAR-T therapies, especially bivalent and bicistronic CAR-T cells. Multi-antigen targeting is a promising means for bypassing the problem of antigen escape, allowing for more precision targeting, reducing the risk for relapse, and perhaps improving survival metrics for patients with R/R B cell lymphomas. It is also important to note limitations: many of the comparisons discussed throughout this manuscript are hypothesis-generating, as there are currently no prospective head-to-head studies comparing the different multi-antigen CAR-T platforms, antigen pairings, or CAR architectures. Although numerous advances have been made in this arena, additional work remains to be done with respect to combinatorial target validation, scalability, optimization of turnaround times, and more effective synthetic immunobiology (70). As we approach the second decade of the commercial availability of CAR-T products, these considerations may help inform future translational science such that CAR-T therapies can result in deep and highly durable remissions in the years to come.
